# The Immunological Basis of Inflammatory Bowel Disease

**DOI:** 10.1155/2016/2097274

**Published:** 2016-12-14

**Authors:** Francesca A. R. Silva, Bruno L. Rodrigues, Maria de Lourdes S. Ayrizono, Raquel F. Leal

**Affiliations:** Inflammatory Bowel Disease Research Laboratory, Gastrocentro, Surgery Department, University of Campinas (UNICAMP), Medical School, Campinas, SP, Brazil

## Abstract

Inflammatory bowel diseases (IBDs) are chronic ailments, Crohn's disease and ulcerative colitis being the most important. These diseases present an inflammatory profile and they differ according to pathophysiology, the affected area in the gastrointestinal tract, and the depth of the inflammation in the intestinal wall. The immune characteristics of IBD arise from abnormal responses of the innate and adaptive immune system. The number of Th17 cells increases in the peripheral blood of IBD patients, while Treg cells decrease, suggesting that the Th17/Treg proportion plays an important role in the development and maintenance of inflammation. The purpose of this review was to determine the current state of knowledge on the immunological basis of IBD. Many studies have shown the need for further explanation of the development and maintenance of the inflammatory process.

## 1. Introduction

Inflammatory bowel diseases (IBDs), notably Crohn's disease (CD) and ulcerative colitis (UC), are widely considered multifactorial diseases and are characterized by chronic intestinal inflammation [1]. These diseases vary according to the affected gastrointestinal area, the depth of the inflammation in the intestinal wall, and the peculiarity of their pathophysiology. The prevalence of IBD is highest in the second to third decade of life with another peak in the 60–70-year-old group [2]. At the onset and during the progression of the disease, associations occur among the genetic factors (which predispose the patient to develop the disease), the environmental factors (which modulate the inflammatory pathways), and the composition of the microbiota [3].

Crohn's disease (CD) is a chronic, transmural, and segmental inflammatory disease. It may affect any part of the gastrointestinal tract, from the mouth to the anus, but is located usually in the terminal ileum. It is characterized by the formation of ulcers, fistulas, stenosis, and intestinal granulomas, with periods of aggravation and remission. Several additional intestinal manifestations may be observed [4]. Ulcerative colitis (UC) is also a chronic inflammatory disease. However, it can affect only the mucosa of the colon and the rectum [5].

The clinical characteristics of IBD are hemorrhagic diarrhea, abdominal pain, tenesmus, urgency to evacuate, anorexia, and weight loss [5, 6]. The etiopathology is not well understood, but environmental factors may be involved, as they predispose genetically susceptible individuals. The severity of the symptoms varies from mild to severe, especially in those who do not respond to the treatment. Patients who do not respond to clinical management and have complications of the disease usually require surgical intervention [7]. The pathophysiology of IBD is not well understood, but there are several hypotheses about its origin: impaired mucosal barrier; dysbiosis; persistent pathogenic infection; and immune deregulation.

## 2. Mucosal Barrier

Patients with genetic susceptibility to IBD are exposed to environmental factors, such as diet and lifestyle, which can induce immune responses that impair the mucosal barrier. The integrity of the epithelial layer enables the intestinal lumen bacteria to communicate with the immune system [8].

The first physical barrier on the mucosal surface is the mucous layer. It is formed by inner and outer layers that are produced by the polymerization of gel-forming mucins secreted by Goblet cells [9]. The inner layer is sterile and the outer is inhabited by commensal bacteria that consume the nutrients in the mucin glycan [9].

**Figure 1:**
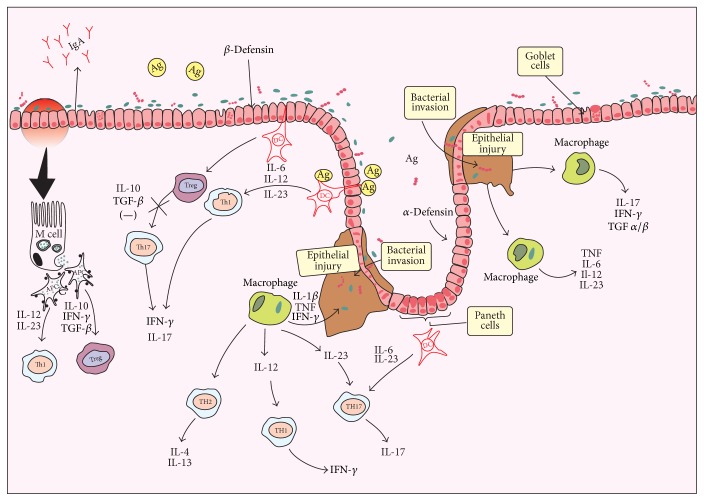
Intestinal epithelial barrier and the immune system in inflammatory bowel disease. Ag: antigen; APC: antigen presenting cells; IL: interleukin; IFN-*γ*: interferon gamma; IgA: immunoglobulin A; M cell: microfold cell; TGF-*β*: transforming growth factor beta; TGF-*α*: transforming growth factor-alpha; Th: T helper cell; Treg: regulatory T cells; TNF: tumor necrosis factor.

The intestinal epithelium is the next barrier and it is considered the second line of defense against bacterial invasion. It comprises enterocytes and specialized epithelial cells called Goblet and Paneth cells [9]. Intestinal epithelial cells (IECs) play a key role in the mucosal barrier, as they prevent the influx of antigens and the invasion by both pathogens and commensal microorganisms [8]. They play a pivotal role in the maintenance of tolerance toward alimentary antigens and commensal microbiota and also activate both innate and adaptive immune responses [10] (Figure 1).

**Figure 2:**
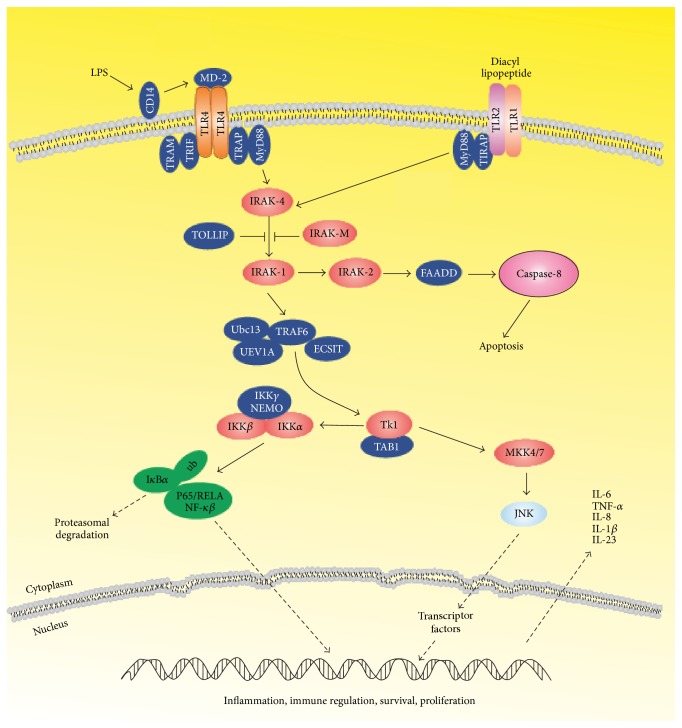
Toll-like receptor signaling pathways. LPS: lipopolysaccharide; CD14: cluster of differentiation 14; MD-2: lymphocyte antigen 96; TLR: toll-like receptor; TRIF: TIR domain-containing adaptor-inducing IFN-*β*; TRAM: TRIF-related adaptor molecule; TRAP: tartrate-resistant acid phosphatase; MyD88: myeloid differentiation primary response 88; IRAK4: interleukin-1 receptor-associated kinase 4; IRAKM: interleukin-1 receptor-associated kinase M; IRAK1: interleukin-1 receptor-associated kinase 1; IRAK2: interleukin-1 receptor-associated kinase 2; TOLLIP: toll interacting protein; FADD: Fas-associated protein with death domain; Caspase-8: cysteine-aspartic protease 8; TIRAP: toll-interleukin-1 receptor domain-containing adaptor protein; UBC13: ubiquitin-conjugating enzyme; TRAF6: TNF receptor-associated factor 6; UEV1A: ubiquitin-conjugating enzyme E2 variant 1A; ECSIT: evolutionarily conserved signaling intermediate In toll pathway; IKK*γ*: nuclear factor kappa-B kinase subunit gamma; IKK*β*: nuclear factor kappa-B kinase subunit beta; NEMO: NF-kappa-B essential modulator; IKK*α*: nuclear factor kappa-B kinase subunit alpha; TK1: thymidine kinase 1; TAB1: TGF-beta activated kinase 1; MKK4/7: mitogen-activated protein kinase kinases 4; JNK: Janus kinase; ub: ubiquinization; ICB*α*: inhibitor of kappa-B; p65/RELA: nuclear factor NF-kappa-B P65 subunit; NF-kB: nuclear factor kappa-B; IL: interleukin; TNF-*α*: tumor necrosis factor-alpha.

To protect the mucosal barrier, the IECs present tight junctions and produce mucins and defensins (*α*-defensins are produced by Paneth cells and *β*-defensins are produced by most of the IECs). IECs also express toll-like receptors (TLR) and nucleotide oligomerization domain receptors (NOD), which are pathogen-sensitive innate immune receptors. IECs then produce chemokines and cytokines to recruit immune cells [8]. Therefore, TLR signaling pathways produce proinflammatory cytokines, such as interleukin- (IL-) 12 and IL-6 by IECs, besides helping to keep the epithelial barrier intact [8, 11]. An impaired epithelial barrier leads to an increased intestinal permeability, which has been observed in CD and also in UC [12]. Some Genome-Wide Association Study (GWAS) suggests that it might represent a primary pathogenetic mechanism in IBD [9]. TLRs belong to the class of transmembrane receptors, called pattern recognition receptors (PRRs), acting as a pro/anti-inflammatory gene activation inducers and control the adaptive immune responses [13, 14]. The TLR family comprises ten different transmembrane receptors that may be found in two locations: in the cell membranes, as is the case with TLR1, TLR2, TLR4, TLR5, and TLR6; into intracellular compartments, such as TLR3, TLR7, TLR8, and TLR9. These genes can be expressed constitutively or inductively along the gastrointestinal tract and in various cell types including enterocytes, Paneth cells, enteroendocrine cells, Goblet cells, myofibroblasts, and subepithelial cells of the intestine immune system, such as monocytes, macrophages, dendritic cells (DC), and CD4 + [15, 16]. In healthy individuals, TLR2 and TLR4 receptors are expressed in smaller amount compared to CD patients, as what triggers a faulty recognition. Environmental, genetic, and immunological factors may alter those receptors [15]. TLR4 is responsible for the recognition of lipopolysaccharide (LPS) and its immune response. The LPS signaling pathway triggers changes in an immunological response, which increases intestinal inflammation [17]. To prevent improper activation against commensal microbiota, TLR is inhibited by cellular mechanisms in the intestinal mucosa. When there is contamination by pathogenic bacteria, the inhibiting TLR mechanism is disabled and positive regulators allow TLR signaling favoring the immune response and the elimination of pathogens [15]. However, the hyperactivation of TLR causes chronic inflammation in IBD. The TLR4 has a significant increase in IEC and in primary mononuclear cells (LPMNCs) of* lamina propria* throughout the lower gastrointestinal tract in IBD patients, which shows the role of this receptor on the mucosal inflammation [15] (Figure 2).

## 3. Microbiota

Although no single agent has been proven to cause IBD, a role for gut microbes has been suspected since the early descriptions of potential infectious pathogens [18]. IBD is clearly associated with intestinal dysbiosis, which is the imbalance in the functions of gut microorganisms that impair host-microbe and immune homeostasis [18]. Human gut contains about 10^11^-10^12^ microorganisms per gram of intestinal lumen content. These microorganisms, called commensal bacteria, can be beneficial to the organism in normal circumstances, as they help to protect the intestinal epithelium [19, 20]. Most of them represent two different phyla, which are the majority of gram-negative bacteria (such as Bacteroidetes) and gram-positive bacteria (such as Firmicutes); the remainder represent a rarer phyla such as Proteobacteria (*Escherichia* and* Helicobacter*) and Actinobacteria; they also include fungi, protists, and viruses [21].

Patients with genetic susceptibility are exposed to environmental factors, such as diet and lifestyle, which can induce immune responses that alter the intestinal microbiota and impair the mucosal barrier [8, 22, 23]. Devkota et al. [23] demonstrated that a diet that does not change the intestinal microbiota is critical to the prevention of IBD. An increase in the incidence of UC was observed in IL-10 deficient mice fed with high levels of saturated fat. This diet promoted the growth of* Bilophila wadsworthia*, a commensal bacterium. This proliferation was probably due to the changes in the composition of bile acid caused by high intake of saturated fat, leading to dysbiosis. von Mutius [24] suggested that the exposure to commensal bacteria during childhood is associated to protection against the development of IBD, for it is critical to stabilize immune tolerance.

In IBD, a dysfunctional interaction between gut microbiota and the mucosal immune system takes place, which may lead to the loss of intestinal immune tolerance by an overreaction of effector T cells that react against common microbial antigens. Thus, there is a decrease of Treg cells that do not properly modulate the effector T cell. This triggers changes in the type and number of microorganisms in the intestinal mucosa, which ultimately leads to an inadequate immune response [25]. Some mouse studies have shown more clearly that the enteric microbiota regulates the development of intestinal immune cell [8]. The balance of some factors, such as TGF-*β* and IL-6, plays a key role in the differentiation of Th17 and Treg [26, 27]. Commensal bacteria can regulate the development of both Th17 and Treg cells suggesting the relevance of local environment induced by commensal microorganisms in immunological homeostasis of gut-associated lymphoid tissues (GALT) [8]. Some other studies highlighted the importance of commensal bacteria for Th17 differentiation in both health and disease: Atarashi et al. [28] demonstrated that commensal bacteria-derived adenosine 5′-triphosphate (ATP) activates a specific subset of colonic* lamina propria* cells, defined as CD70^high^CD11c^low^ DCs, which leads to Th17 cells differentiation. In response to ATP stimulation, this subset expresses Th17-prone molecules, such as IL-6 and IL-23p19, and induces Th17 differentiation of cocultured naive CD4+ T cells. Ivanov et al. [29] reported that a small commensal intestinal microbiota, segmented filamentous bacterium (SFB), is sufficient to induce Th17 cells in the intestinal* lamina propria*.

One of the many mechanisms that affects host inflammatory responses is associated with short-chain fatty acids (SCFA). Their levels are significantly decreased in IBD; it may be a key factor compromising both intestinal and immune homeostasis [30]. Atarashi et al. [28] demonstrated that SCFA-producing bacterial strains in* Clostridia* clusters IV, XIVa, and XVII from a healthy human fecal sample induced colonic regulatory T (Treg) cell differentiation, its expansion, and function.

In IBD, B cell responses also occur: IgA is a major class of immunoglobulin produced in the mucosa, including the gut. In the intestinal lumen, IgA is produced as polymeric IgA at high concentrations, which is transported by the polymeric immunoglobulin receptor (pIgR) expressed on IECs and released into the intestinal lumen as secreted IgA (SIgA). SIgA covers antigens in order to inhibit their binding to the host epithelium and, therefore, the penetration into the* lamina propria* [31, 32]. The binding of IgA to the commensal* Bacteroides thetaiotaomicron* inhibits innate immune responses by impairing bacterial gene expression [33].

Mononuclear phagocytes, such as macrophages and DCs, are responsible for the lack of immunological response to commensal bacteria, which is relevant to maintaining gut homeostasis [31, 32]. The microbiota is important for the production of pro-IL-1*β* and the precursor of IL-1*β*, in resident mononuclear phagocytes. When the epithelial barrier is intact, commensal bacteria cannot induce the maturation of pro-IL-1*β* into biologically active mature IL-1*β* and thus a state of low response is maintained [32]. By contrast, enteric pathogens, such as* Pseudomonas aeruginosa *and* S. Typhimurium*, may induce the maturation of pro-IL-1*β* as it activates caspase-1 via the NLRC4 (NOD-, LRR-, and CARD-containing 4) [32]. Microbiota also promotes immune response by the production of IL-22 by innate lymphoid cells (ILCs) [34]. A study with germ-free mice reported an impaired gut IL-22 production, suggesting that there may be a requirement for commensal bacteria or their metabolites [35]. Mice with impaired cells that express IL-22 showed an increase in the susceptibility to infection by* C. rodentium*, which suggests that commensal bacterial-driven IL-22 produced by ILC3s is important for protection against infectious pathogens [34, 36, 37].

## 4. Innate Immunity

Innate immunity is the first defense against invading microorganisms and other harmful agents. Innate response is activated minutes after the invasion by microorganisms. It may last a few hours and has no immunological memory [38]. The tissues affected by IBD present activated macrophages, which also express the CD14 monocyte marker (cluster differentiation 14) and they are phenotypically heterogeneous, unlike what is observed in the normal gut.

Macrophage cells can eliminate specific pathogens, such as peptides and lipopolysaccharides using free radicals and proteases. Cell membrane histocompatibility complex is responsible for specific pathogen-associated antigen. After formation of this complex, T cells are presented to the antigens located on the surface receptors [39]. During an IBD acute phase, the number of macrophages in the intestinal mucosa increases dramatically. In this process, macrophages express large number of T cells and costimulatory molecules such as CD40, CD80, and CD86, involved in the inflammatory process.

In nonpathogenic conditions, macrophages are limited by the intestinal mucosal microenvironment. They present noninflammatory phenotypes that are decoded by a decreased expression of receptors related to innate immunity activation. Therefore, a limited production of proinflammatory cytokines, such as interleukin- (IL-) 1*α* and IL-1*β*, and tumor necrosis factor-alpha (TNF-*α*) is observed [40, 41].

Another cell type involved in this process is dendritic cells (DC), which are antigen presenting cells (APC). They are directly related to local immune regulation. In both CD and UC, DCs are activated in small numbers but have strongly expressed microbial receptors. This causes an overexpression of some proinflammatory cytokines, such as IL-6 and IL-12 [42]. DCs transport antigens to the gut-associated lymphoid tissue (GALT) where the naive T cells are activated. They can determine whether there will be an immune response or not. Due to TLR, DCs can recognize certain molecular structures of the bacteria, such as the PAMP (pathogen-associated molecular pattern), and it enables them to distinguish very similar microorganisms. Because of these functions, DCs became fundamental in IBD, as they are responsible for the balance between the tolerance to commensal microorganisms and immune activity [43].

In healthy patients, TLR signaling helps to protect the epithelial barrier and assists tolerance to commensal bacteria. However, malfunction in TLR signaling can induce an intestinal inflammatory response with different clinical phenotypes, including the IBD [43]. A major target of the TLR signaling is the activation of transcription factor NF-kB [44], which regulates the expression of a variety of genes responsible for controlling the innate response, such as IL-1, IL-2, IL-6, IL-12, and TNF-*α* [45, 46]. Both IL-1 and TNF-*α* share numerous proinflammatory property responsible for the development of IBD [47, 48].  Table 1 shows the main cytokines involved in innate immune response.

## 5. Adaptive Immunity

Adaptive immunity presents an important role in the pathogenesis of the disease. T cells regulate the immune response in IBD. They proliferate in the peripheral blood and differentiate when they are stimulated by the presence of antigens. The main subtypes of T helper (Th) cells are Th1, Th2, Treg, and Th17. Each of these subtypes has relevant immune functions. For example, Th1 eliminates pathogenic agent present in the cells; Th2 controls allergic reactions and protects the body from parasites; Th17 among all its functions are to remove the extracellular bacteria and fungi; Treg cells are to promote tissue repair. However, alterations in the proliferation of T cells and their subsets may have an excessive increase of chemokines and cytokines, leading to the worsening or maintenance of the mucosal inflammatory process [49].

After the identification of antigens in gut-associated lymphoid tissue (GALT), the activation of effector CD4+ and CD8+ T cells (Th1 and Th2) occurs, as well as the maturation of B lymphocytes that produce antigen-specific immunoglobulins. T cells in contact with IFN-*γ* differentiate into Th1 cells. Th1 cells are responsible for secrete different types of proinflammatory cytokines, such as IL-1, IL-2, IL-6, IL-8, IL-12, TNF-*α*, and IFN-*γ* [49]. IFN-*γ* is responsible for macrophage activation. Studies in mouse models in which CD was induced by trinitrobenzene sulfonic acid and IFN-*γ* expression were increased in the local intestinal mucosa and in the spleen [50]. The antigen presenting cells secreting IL-4 act on the Th cells surface receptors activating STAT-6, which promotes the differentiation into Th2 cells [49]. IL-13 and TNF-*α* act on Th2 surface receptor activating and promoting the proliferation of this cell type [49]. The increase of Th2 is simultaneous to the increase of IL-5 and IL-13 in the UC inflamed mucosa [51]. Th2 cells secrete IL-4, IL-5, IL-9, and IL-13, which regulate the differentiation and activation of B cells [52–54]. These two cell types also secrete TNF-*α*, a Treg suppressor. However, Th1 cells secrete them in higher amounts. Breese et al. [55] observed that there is a higher increase of secretion of IFN-*γ* in CD than in UC, and Fuss et al. [56] observed a higher expression of IL-5 in UC than in CD. Therefore, Th1 and Th2 cells are essential in the development of intestinal inflammation. This response is firstly induced by IL-12 produced from active DC and is mediated by an excessive IFN-*γ* production [57, 58]. The balance between Th1 and Th2 occurs when the released cytokines inhibits the action of another Th cell, as for example, IFN-*γ* secreted by Th1 cell inhibits proliferation of Th2 cells, while IL-4, IL-10, and IL-13 secreted by Th2 cells inhibit exacerbated responses of Th1 cell [49]. Thus, the imbalance of Th1/Th2 subsets is directly involved in the pathogenesis of several autoimmune and immune-mediated diseases and inflammatory diseases, and they have fundamental performance in the development and maintenance of inflammation in IBD [59]. Proinflammatory cytokines as IL-1, IL-2, IL-6, and IL-8, which are secreted by Th1, are associated with cellular immune responses and anti-inflammatory cytokines as IL-4, IL-10, and IL-13, which are secreted by Th2 cells, are directly involved in humoral immune response. The balance between proinflammatory and anti-inflammatory properties is determined by the Th1/Th2 cells ratio, determining the types of immune responses that patients develop [49]. Therefore, many researchers have been studied cytokines and T cells subtypes to discover new targets for the IBD treatment [60].

Moreover, APCs produce IL-12, which induces the expression of IFN-*γ* by Th1 cells, besides IL-2 and TNF-*α*. Th2 cells produce IL-4, which stimulates the production of IL-5 and IL-10 [61]. Th1 cells increase the expression of MHC-II molecules (Major Histocompatibility Complex II) in the APC, which activates CD8+ T cells and macrophages [62]. The progression of CD is mainly mediated by CD4+ Th1 and Th17 cells, and IFN-*γ* is one of the main cytokine expressed in this disease [51]. The antigen presentation mediated by MHC-II is fundamental to develop a CD4+ T cell immune response [63]. The MHC-II molecule is primarily expressed on mature APCs, which leads to the activation of effector T cell and FoxP3+ Treg cell [64]. Due to MHC-II antigen presentation machinery, IECs are able to process and present intestinal luminal antigens [65]. Thelemann et al. [63] reported that mice with MHC class II depletion specifically in IECs have increased innate immune cell infiltration and proinflammatory cytokines. Besides, they presented Th1 response with similar levels of Th17 cells compared to wild littermates. In contrast, mice presenting MHC class II depletion in innate lymphoid cells type 3 (ILC3s) have increased Th17 cell numbers compared to control group [63]. The results of these studies suggest that ILC3s limit Th17 differentiation through the expression of MHC-II by an unknown mechanism and highlight the multiple capable of cell type's antigen presentation and T cell differentiation [66, 67].

In the immunological responses described above, one that stands out in the CD development process is the activation of IL-23/IL-17 response in the target tissues [69], in addition to the Th1 response. IL-23 is produced by APC, DC, and macrophages, and it stimulates the production of IL-17, TNF-*α*, and IL-6 by Th17 cells [61]. IL-17 presents a proinflammatory activity, which induces the production of cytokines that increase Th1 response; chemokine expression; adhesion molecules by epithelial and endothelial cells; fibroblast proliferation; and growth factor expression, such as G-CSF (Granulocyte Colony Stimulating Factor) and GM-CSF (Granulocyte Colony Macrophage Stimulating Factor) [68].  Table 2 shows the main cytokines involved in adaptive immune response.

Humoral immunity is also changed, and B cells produce and secrete a deregulated amount of antibodies, especially IgG, IgM, and IgA [70]. In CD, the IgG-1, IgG-2, and IgG-3 levels are high both in serum and in the intestinal mucosa, compared to healthy subjects [71]. Several autoantibodies and antibodies against specific microorganisms were identified in IBD [72]. The best known are the neutrophil cytoplasmic antibody (ANCA) and the antibody against* Saccharomyces cerevisiae *(ASCA). ANCA autoantibody production is triggered by bacterial antigens. It is present in 65 to 70% of patients with UC and constitutes one of the few markers for the disease, as the other antibodies are more efficient markers for CD. The ASCA antibody is positive in 55 to 70% of CD patients. Other antibodies are OmpC, I2, CBir1-flagellin, A4-Fla2 flagellin and Fla-X. OmpC originates from an antigen of the membrane surface proteins of the bacteria* E. coli*. In contrast, I2 reacts against* P. aeruginosa*, while CBir1-flagellin antibody is directed against flagella of commensal bacteria [73, 74]. The A4Fla2 and Fla-X flagellins have been recently discovered and some CD patients are seropositive. In a prospective study evaluating 252 patients with CD, 59% were positive for A4-Fla2 and 57% for Fla-X, while 76% of the overall sample had localized disease in the small intestine [75]. Another study showed that patients undergoing ileal pouch anal anastomosis for UC with positive ASCA IgG and CBir-1 were related to the development of fistulas and CD in the ileal pouch. The identification of this group of patients with a high risk of complications may allow early and more aggressive measures to prevent ileal pouch failure [76].

More recently, studies have evaluated anti-glycan antibodies, which act against saccharide components of the cell membrane of microorganisms (bacteria, fungi and viruses). These antibodies are found in a variable percentage of patients with CD (10–28%, except for g-ASCA, whose sensitivity is higher, 46–60%). The most well-known antibodies are: anti-*Saccharomyces cerevisiae* antibody (gASCA), anti-laminaribioside carbohydrate antibody (ALCA), anti-chitobioside carbohydrate antibody (ACCA), anti-mannobioside carbohydrate antibody (AMCA), anti-laminarin IgA (anti-L), and anti-chitin IgA (anti-C). Besides aiding in the diagnosis of CD, these markers may predict disease progression. For example, gASCA and AMCA are signs of short duration disease. gASCA and ALCA are biomarkers of disease at a young age, and ACCA suggests long-term illness, while anti-L and anti-C indicate colonic involvement. Although the sensitivity is not high for all these markers, the specificity is slightly higher (about 40%) [77]. The findings on anti-glycan antibodies suggest a connection between the innate and adaptive immune systems. This reflects the loss of tolerance to commensal microorganisms, which is considered a hallmark of the immunopathogenic process in IBD.

## 6. T Regulatory Cells

Treg cells are cells capable of inhibiting other Th subtypes, such as Th1, Th2, and Th17 through the release of cytokines IL-10 and TGF-*β* and by direct contact with the surface of Th cell [49]. Tregs have as their main characteristic a specific surface marker called Foxp3, which distinguishes it from other Th subtypes. These cells are subdivided into two main categories: natural regulatory T cells (nTreg) and induced regulatory T cells (iTreg). The nTreg cells are able to suppress autoimmune diseases and immune responses, and they induce immunological tolerance [49]. The reduction of Treg cells is associated with IBD pathogenesis [78, 79]. Effector T cells may be suppressed through cytokines produced by T regulatory (Treg) cells, which are extremely important for maintaining of the intestinal mucosa homeostasis. They are enrolled in the suppression of the immune responses against an exacerbated number of bacteria. This occurs due to the production of anti-inflammatory cytokines such as IL-10 and TGF-*β* [80] (Figure 1).

In an experimental study, naive T cells without CD4+ and CD25+ Treg cells were injected into mice with T cell defection. High response to intestinal symbiotic bacteria was verified, which led to the development of an autoimmune colitis [81]. However, when T cells with CD4+ and CD25+ Treg cells were injected into mice models that presented IBD pathological injuries, these cells were recruited to the intestinal lymphatic tissues and to* lamina propria*. They then migrated to the spleen to exert an immune regulation [82].

Tregs perform a huge anti-inflammatory action, as was verified in an experimental study of UC. However, these cells were lacking in the peripheral blood of patients with the active disease, when compared to those who were in the inactive phase or in the control group [83–85]. For Tregs to be functional, a signal made by TGF-*β* is needed. However, this signal is weakened in IBD due to the upregulation of an inhibitory molecule called Smad7. Fantini et al. [86] observed that the* lamina propria* effector T cells of IBD patients do not respond to Treg signaling. This finding was reversed by the presence of an antisense oligonucleotide anti-Smad7. Therefore, a possible inhibition of Treg cells can contribute to the development of IBD [9].

Reductions of Treg cells were found in peripheral blood and colonic mucosa in IBD patients, suggesting that lower expression of Treg cells is associated with IBD pathogenesis [78, 79].

## 7. T Helper 17 Cells

For the differentiation and proliferation and Th17 cells, IL-23 act on the IL-23 receptor on the surface of Th cells and activate cytoplasmic signal transduction and transcriptional activation factor 3 (STAT-3). This activation occurs in the presence of TGF-*β*, IL-6, or IL-21 [49]. The Th17 cells are activated when several cytokines such as IL-17, IL-21, and IL-22 are released. Some clinical studies have found high levels of Th17 and IL-17 in mucosa of IBD patients compared to healthy controls. Th17 cells are mainly distributed in the* lamina propria* of the UC intestinal mucosa and in the submucosa and muscle layer of the mucosa of CD patients [87]. IL-17 is directly associated with the release of proinflammatory factors and also responsible for the induction of immune cell transfer to peripheral tissues. After this process, IL-17 binds to the surface receptors and finally activates NF-kB, releasing proinflammatory factors [49]. It has been observed in studies that showed high IL-17 serum expression in the IBD patients [88].

Th17/Treg cells remain in balance under normal conditions; however, this balance can be disrupted due excessive increases of Th17 cells and decrease of Tregs, leading to damage to the intestinal mucosa [49]. T cells differentiate into Th17 in the presence of IL-6 and low TGF-*β* concentrations, thereby inhibiting proliferation of Treg cells. On the other hand, high concentrations of TGF-*β* inhibit Th17 production and increase Treg production [89]. Th17 is increased in the peripheral blood of IBD patients, while Treg cells are decreased, suggesting that the Th17/Treg proportion plays an important role in the development and maintenance of inflammation [49].

## 8. Intestinal Fibrosis and the Inflammatory Process

Intestinal fibrosis is commonly characterized as an excessive deposition of extracellular matrix (ECM), resulting from chronic inflammation and impaired intestinal wound healing [90]. Inflammation process is necessary for the development of intestinal fibrosis [91]. However, in vivo and in vitro studies suggest that fibrogenic mechanisms can be distinct from the inflammation process. Particularly, in IBD, it is difficult to distinguish the inflammatory response from the fibrotic process, because the cells responsible for each response are intimately associated in the mucosa microenvironment [90].

The main mechanism responsible for the formation of intestinal fibrosis is the growth and increase of the fibroblast population [90]. In IBD, isolated fibroblasts show a faster proliferation rate compared to a non-IBD normal mucosa [92, 93]. In support of this fact, intestinal fibroblasts can increase their growth rate in vitro conditions similar to the inflamed gut [90]. These conditions can activate molecules, such as platelet-derived growth factor (PDGF), insulin like growth factor I (IGF-I), epithelial growth factor (EGF), basic fibroblast growth factor (bFGF), and connective tissue growth factor (CTGF). They also induce the production of proinflammatory cytokines, such as IL-1*β*, IL-6, and TNF-*α* [90, 92, 94, 95]. After the fibroblasts are recruited, they must be retained at the inflammatory site. This action is mediated by proinflammatory cytokines, such as TNF-*α* and IFN-*γ*, and both of them can lead to the fibroblasts' migration in vitro [96]. How much this reduction of the migratory capacity in vivo contributes to the development of fibrosis in IBD is unclear [90]. Therefore, the fibroblasts may also contribute to the intestinal inflammatory conditions in IBD, mainly in CD patients, who are prone to develop fibrostenosis.

## 9. Conclusion

The immunological aspects of IBD, specifically CD and UC, involve impaired innate and adaptive responses which may be associated with genetic susceptibility, environmental factors, and intestinal microbiota. Th17 cells play an important role in the development and in the maintenance of the disease. Besides, defective anti-inflammatory mechanisms, such as the decrease of Treg cells, are also involved in maintaining the ailment. Moreover, the understanding of the exclusive role of immune cells in all of this process has changed in face of new discoveries, since IECs are also relevant cells in IBD.

## Figures and Tables

**Table 1:** Main cytokines of the innate immune response, cells that produce them, and the principle actions.

Cytokines	Types of cells	Main functions in IBD
IL-1	Monocytes Epithelial cells Macrophages Endothelial cells	Activating T cells to produce IL-8 and IL-6 Development of IBD [47–49]

IL-6	Macrophages Endothelial cells Fibroblasts	Playing a key role in the differentiation of Th17 and Treg cells, in balance with some factors, such as TGF-*β* [26, 27, 49]

IL-12	Macrophages Dendritic cells	Promoting the differentiation of Th1 cells [61]

IL-23	Macrophages	Stimulating the production of IL-17, TNF-*α*, and IL-6 [61]

TNF-*α*	Macrophages Dendritic cells Endothelial cells	Acting on Th2 surface receptor promoting the proliferation of this cell type [49] Inhibiting Treg cells [52–54]

**Table 2:** Main cytokines of the adaptive immune response, cells that produce them, and the principle actions.

Cytokines	Types of cells	Main functions in IBD
IL-2	T cells	Inducing proliferation of T and B cells and the production of IFN-*γ* [49]

IL-4	Th2 cells Mast cells	Promoting the differentiation of Th2 cells Inhibiting exacerbated responses of Th1 cells [49]

IL-10	Macrophages Dendritic cells Treg	Inhibiting exacerbated responses of Th1 cell [49]

IL-17	Th17 cells Neutrophils	Promoting inflammation by inducing the production of IL-6, IL-1, and TNF-*α* Inducing the production of cytokines that increase Th1 response Inducing chemokine expression, adhesion molecules by epithelial and endothelial cells, fibroblast proliferation, and growth factors expression, such as G-CSF and GM-CSF [68]

TGF-*β*	T cells Macrophages Fibroblasts	Inhibiting Th subtypes, such as Th1, Th2, and Th17 cells [49] Playing a key role in the differentiation of Th17 and Treg, in balance with some factors, such as IL-6 [26, 27]

IFN-*γ*	Th1 cells TCD8^+^ cells NK cells	Activation of macrophages [50] Inducing the production of IL-12 [57, 58]
